# Interlaboratory comparison of molecular detection of *Cyclospora cayetanensis* in routine diagnostic laboratories

**DOI:** 10.1371/journal.pone.0361578

**Published:** 2026-10-28

**Authors:** Jacky Flipse, Angelino T. Tromp, Esmée Ruizendaal, Els Wessels, Fleur M.H.P.A. Koene, Jaap J. van Hellemond, Jaco J. Verweij, Linda J. Wammes, Noortje van Maarseveen, Raymond Steen, Rob Schuurman, Theun de Groot, Thomas J. Roodsant, Wouter Bekers, Guido J.H. Bastiaens

**Affiliations:** 1 Laboratory for Medical Microbiology and Immunology, Dicoon, Elst, The Netherlands; 2 Department of Medical Microbiology and Immunology, Rijnstate, Arnhem, The Netherlands; 3 Laboratory for Medical Microbiology, Medical Laboratory Services, Willemstad, Curaçao; 4 Department of Medical Microbiology, RadboudUMC, Nijmegen, The Netherlands; 5 LUCID-M, Leiden University Medical Centre, Leiden, The Netherlands; 6 Department of Medical Microbiology and Infectious Diseases, Erasmus MC University Medical Center, Rotterdam, The Netherlands; 7 Microvida, Laboratory for Microbiology and Immunology, Elisabeth-Tweesteden Hospital, Tilburg, The Netherlands; 8 Laboratory for Medical Microbiology, Unilabs, Amsterdam, The Netherlands; 9 Department of Medical Microbiology, University Medical Center Utrecht, Utrecht, The Netherlands; 10 Centre for Infectious Disease Control, National Institute for Public Health and the Environment, Bilthoven, The Netherlands; University of Alcala Faculty of Medicine and Health Sciences: Universidad de Alcala Facultad de Medicina y Ciencias de la Salud, SPAIN

## Abstract

**Objectives:**

Although molecular detection of *Cyclospora cayetanensis* is increasingly incorporated into multiplex gastrointestinal diagnostic workflows, dedicated external quality assessment (EQA) programmes for *Cyclospora* remain limited. We performed a nationwide interlaboratory comparison to evaluate routine molecular detection of *C. cayetanensis* across decentralized ISO15189-accredited laboratories in the Netherlands.

**Methods:**

An anonymized 11-sample panel containing unpreserved stool specimens and eNAT-preserved stool suspensions was distributed to laboratories in the Netherlands (n = 8) and Curaçao (n = 1). Laboratories analysed samples using their routine diagnostic workflows. Workflow characteristics, DNA extraction procedures, molecular platforms, and interpretation criteria were collected by a structured questionnaire. Because detection of *Cryptosporidium* spp. is usually performed within the same workflow and present similar extraction challenges, *Cryptosporidium*-positive samples were included as internal workflow controls. Aggregated retrospective laboratory data were collected to explore surveillance feasibility and to gain insight in seasonality of *C. cayetanensis* and *Cryptosporidium* spp.

**Results:**

Seven laboratories performed molecular detection of *C. cayetanensis*, while all nine performed detection of *Cryptosporidium* spp. Complete qualitative agreement was observed for *Cryptosporidium* spp. detection. For *C. cayetanensis*, overall qualitative agreement was high, although one false-positive and three false-negative results occurred among core samples. Detection of a low-positive educational sample varied substantially between laboratories, indicating reduced reproducibility near assay-specific limits of detection. This study shows that eNAT-preserved stool suspensions remained stable under ambient transport conditions. Retrospective laboratory data showed low-level year-round *C. cayetanensis* detection and a seasonal increase in *Cryptosporidium* spp. positivity during late summer and autumn.

**Conclusions:**

Routine molecular detection of *C. cayetanensis* demonstrated good overall interlaboratory agreement across decentralized diagnostic laboratories. However, reproducibility decreased for low-positive samples, highlighting the importance of assay validation, external quality assessment, and cautious interpretation of weak positive *C. cayetanensis* PCR results.

## Introduction

*Cyclospora cayetanensis* is an intestinal coccidian parasite associated with foodborne and waterborne gastrointestinal disease. Infection typically causes prolonged or relapsing watery diarrhoea and may result in substantial morbidity, particularly when diagnosis and appropriate treatment are delayed. Although cyclosporiasis is more common in tropical and subtropical regions, cases in Europe occur predominantly among travellers and through foodborne transmission, including outbreaks associated with imported fresh produce [1]. Increasing implementation of multiplex gastrointestinal molecular diagnostics has likely increased detection in routine clinical microbiology laboratories. Accurate detection is therefore relevant for individual patient management, outbreak recognition, and public health surveillance.

Real-time PCR has largely replaced microscopy for routine detection of intestinal protozoa because of improved analytical sensitivity, higher throughput, and compatibility with syndromic gastrointestinal testing [2–4]. However, molecular workflows can differ substantially, including specimen processing, DNA extraction, PCR targets, primer and probe sequences, amplification platforms, and interpretation criteria [5–7]. Such heterogeneity may be particularly relevant for *C. cayetanensis*, where low oocyst density, intermittent shedding, and limited assay standardization can affect reproducibility near the analytical limit of detection [8].

*Cryptosporidium* spp.-positive samples were included because *Cryptosporidium* spp. and *C. cayetanensis* are commonly detected within the same gastrointestinal molecular workflows [2,4,9–11] and because oocysts of both parasites have robust outer walls that require effective disruption for efficient DNA recovery, thereby making pre-analytical processing an important determinant of analytical performance [5,12]. In the present study, *Cryptosporidium* spp.-positive samples therefore served as internal workflow controls, allowing assessment of general workflow performance alongside *C. cayetanensis*-specific assay performance [6].

Participation in EQA programmes is an essential component of ISO15189-accredited medical microbiology laboratories. While EQA schemes exist for several intestinal protozoa, dedicated quality assessment for molecular detection of *C. cayetanensis* remains limited. Consequently, little is known about the reproducibility of routine *C. cayetanensis* molecular diagnostics across decentralized laboratory networks.

The primary objective was to perform an interlaboratory comparison for routine molecular detection of *C. cayetanensis* in the Netherlands and Curaçao. Secondary objectives were to evaluate workflow robustness using *Cryptosporidium* spp. samples, assess the feasibility of eNAT-preserved stool suspensions for decentralized sample distribution, and explore retrospective laboratory data for surveillance purposes.

## Materials and methods

### Study design and participating laboratories

The Laboratory for Medical Microbiology and Immunology, Dicoon (Elst, the Netherlands), coordinated the interlaboratory comparison. Nine laboratories participated: eight in the Netherlands and one in Curaçao.

Participating laboratories received an anonymized sample panel and a structured questionnaire covering diagnostic algorithms, extraction workflows, sample input and elution volumes, molecular platforms, PCR targets, Ct interpretation criteria, annual test volumes, and positivity rates. Workflows are described in S1 Table.

Qualitative agreement between laboratories was the primary outcome measure. Ct values were analysed descriptively only because direct quantitative comparison between heterogeneous workflows is limited by assay-specific analytical characteristics and pre-analytical variability. Exploratory Ct normalization analyses are provided in supplementary material (S2A-C Tables).

### Interlaboratory comparison panel

Residual stool samples from routine diagnostics were included after anonymization if they had tested positive for *C. cayetanensis* or *Cryptosporidium* spp. by PCR and residual material was available for research use.

The panel consisted of 11 specimens: eight stool suspensions at approximately 100 mg/mL (2 pea sized scoops in 2mL eNAT medium (#606C, Copan, Italy)) and three unpreserved stool samples. Sample number 1, 2 and 8 were unpreserved stool samples, whereas sample number 3–7 and 9–11 were stool suspensions in eNAT. Samples were selected to represent Ct values observed in routine diagnostics (S1A and S1B Figs for details). The panel included positive and negative specimens for both parasites and one low-positive educational *C. cayetanensis* sample designed to challenge detection near the assay limit of detection. Laboratories were instructed to process native stool samples according to their routine clinical workflow and eNAT suspensions as ready-to-test specimens.

### Use of eNAT and stability assessment

To assess stability during distribution, the coordinating laboratory retested eNAT-preserved samples before shipment and after simulated ambient transport conditions (S1 Table). Additional validation experiments assessed stability of gastrointestinal protozoa in eNAT during prolonged room-temperature storage (S2 Fig). The validation experiments showed high degree of homogeneity in eNAT-preserved stool suspensions (Range of Ct values 0.05–3.09, average 1.31Ct).

### Retrospective laboratory data

Participating laboratories were invited to provide aggregated retrospective laboratory data from the preceding 12 months, including numbers of stool samples analysed and positivity rates for *Cryptosporidium* spp. and *C. cayetanensis*.

### Statistics

Descriptive analyses were performed using GraphPad Prism version 10.4.0. Because of workflow heterogeneity and limited sample size, formal quantitative comparison between workflows was not considered appropriate.

### Data availability

All relevant data are within the paper and supporting information files.

### Ethics statement

Clinical specimens were obtained during routine microbiological diagnostics and anonymized before use in this study. Patients had not objected to the secondary use of residual diagnostic material. Under applicable Dutch regulations, research using anonymized residual diagnostic material of this nature does not require review under the Dutch Medical Research Involving Human Subjects Act (WMO); therefore, no formal Medical Ethics Review Committee (METC) approval or waiver was obtained. No additional patient interventions were performed.

### Inclusivity in global research

Additional information regarding the ethical, cultural, and scientific considerations specific to inclusivity in global research is included in the Supporting Information (S1 File).

## Results

### Participating laboratories and workflows

Nine laboratories participated, including eight laboratories in the Netherlands and one laboratory in Curaçao. All participating laboratories routinely performed molecular detection of *Cryptosporidium* spp., while seven laboratories routinely performed molecular detection of *C. cayetanensis*. Across the ten molecular workflows used by the nine participating laboratories, pre-analytical processing included mechanical disruption (n = 2), enzymatic lysis (n = 1), and non-mechanical stool-suspension-based processing (n = 7). Detection was performed using five commercial syndromic or multiplex PCR workflows and five laboratory-developed real-time PCR workflows (S1 and S3 Tables). Workflow-specific interpretation thresholds are summarized in S1 Table where available. For commercial platforms without user-accessible Ct values, results were interpreted according to the manufacturer-defined qualitative criteria..

### Panel stability

Retesting by the coordinating laboratory demonstrated minimal Ct variation during simulated ambient transport conditions, generally remaining within 1 Ct value after 7 days of storage at room temperature. Additional validation experiments demonstrated stability of gastrointestinal protozoa in eNAT-preserved suspensions for up to 8–11 days at room temperature (S2 Fig).

### Molecular testing of *Cyclospora cayetanensis* and *Cryptosporidium* spp

*Cyclospora cayetanensis* and *Cryptosporidium* spp*.* PCR results are summarised in Table 1. Molecular diagnostics for *C. cayetanensis* and *Cryptosporidium* spp*.* were performed by seven and nine laboratories, respectively. One laboratory could not participate in two samples (number 9 and 11).

**Table 1:** Qualitative performance of molecular diagnostic assays for *Cyclospora cayetanensis* and *Cryptosporidium* spp. by the participating laboratories.

Sample#		1	2	3	4	5	6	7	8	9	10	11
Specimen												
unpreserved stool		**x**	**x**						**x**			
stool suspension in eNAT^ǂ^				**x**	**x**	**x**	**x**	**x**		**x**	**x**	**x**
Intended result												
*Cyclospora cayetanensis*		**+**	**+**	**+**	**+**	**–**	**+**	**+**	**+**	**–**	**–**	**+**
*Cryptosporidium* spp.		**–**	**–**	**–**	**–**	**+**	**–**	**–**	**–**	**+**	**+**	**–**
*Cyclospora cayetanensis*												
Participating laboratories		**7**	**7**	**7**	**7**	**7**	**7**	**7**	**7**	**6**	**7**	**6**
Reported result	Correct	7	7	7	6	6	7	6	6	6	7	2*
	Incorrect **	–	–	–	1^N^	1^P^	–	1^N^	1^N^	–	–	4^N^
	% correct	100	100	100	86	86	100	86	86	100	100	33
Core (C) or Educational (E)		C	C	C	C	C	C	C	C	C	C	E
*Cryptosporidium* spp.												
Participating laboratories		**9**	**9**	**9**	**9**	**9**	**9**	**9**	**9**	**8**	**9**	**8**
Reported result	Correct	9	9	9	9	9	9	9	9	8	9	8
	Incorrect	–	–	–	–	–	–	–	–	–	–	–
	% correct	100	100	100	100	100	100	100	100	100	100	100
Core (C) or Educational (E)		C	C	C	C	C	C	C	C	C	C	C

ǂstool suspension, prepared by suspending unpreserved stool in eNAT at ~100 mg/mL.

*Two laboratories technically detected *C. cayetanensis* DNA in the educational sample (# (11), however the Ct value exceeded the cut-off of that laboratory (Ct 35.0), thus the respective laboratory would have reported the sample as negative. One laboratory did not participate for this sample for lack of material.

**None of the participating laboratories detected >1 incorrect result in a core sample.

Superscript next to incorrect results denotes whether the incorrect result concerned a false-positive (P) or false-negative result (N).

Seven laboratories performed molecular detection of *C. cayetanensis*. Overall qualitative agreement for core samples was high, with most laboratories correctly identifying positive and negative specimens (Table 1; 65/69 agreement on core results (94.2%)). One false-positive (1/69; 1.4%) and three false-negative results (3/69; 4.3%) were observed among the ten core sample results. None of the participating laboratories generated more than one discrepant result among the core samples. Observed discrepancies were predominantly associated with samples generating high Ct values during reference testing (Table 1, S2A-B Tables). Both commercial assays and laboratory-developed workflows demonstrated acceptable qualitative performance overall; however, analytical sensitivity and interpretation thresholds differed between workflows [6,7].

Detection variability increased substantially for low-positive samples near assay-specific limits of detection. Detection of the educational sample varied substantially between laboratories, with several workflows failing to detect the sample or generating Ct values exceeding local reporting thresholds (Table 1).

### Microscopy for *Cyclospora cayetanensis*

Four laboratories reported availability of microscopy for *C. cayetanensis*. However, microscopy was rarely used in routine diagnostics and reported sensitivity relative to PCR was limited, particularly for low-positive samples. Compared with PCR, the reported sensitivity of microscopy was 0% (0/5), 33% (3/9), and 50% (3/6) for samples with Ct ≤ 38, whereas no samples with Ct > 38 were detected by microscopy (0/19).

### Performance for *Cryptosporidium* spp

All nine participating laboratories correctly identified all *Cryptosporidium*-positive and -negative samples, demonstrating complete qualitative agreement across heterogeneous workflows (Table 1). No false-positive or false-negative *Cryptosporidium* spp. results were observed.

Complete agreement (97/97; 100%) was achieved despite substantial methodological heterogeneity, including differences in specimen input, extraction chemistry, primer and probe sequences, amplification platforms, and interpretation thresholds. Both commercial assays and laboratory-developed PCRs demonstrated robust qualitative performance.

Although qualitative agreement was complete, considerable variation in Ct values (>9Ct per sample) was observed between workflows, reflecting differences in sample processing, extraction efficiency, amplification chemistry, and platform-specific analytical characteristics (S1 and S3 Tables).

### Retrospective laboratory surveillance data

Six laboratories provided retrospective laboratory data for *Cryptosporidium* spp. and five provided data for *C. cayetanensis*. *C. cayetanensis* positivity remained low throughout the study period without a distinct seasonal distribution (Fig 1A). In contrast, *Cryptosporidium* positivity demonstrated a seasonal increase between August and November 2024 in both hospital and first-line diagnostic populations (Fig 1B).

**Fig 1:**
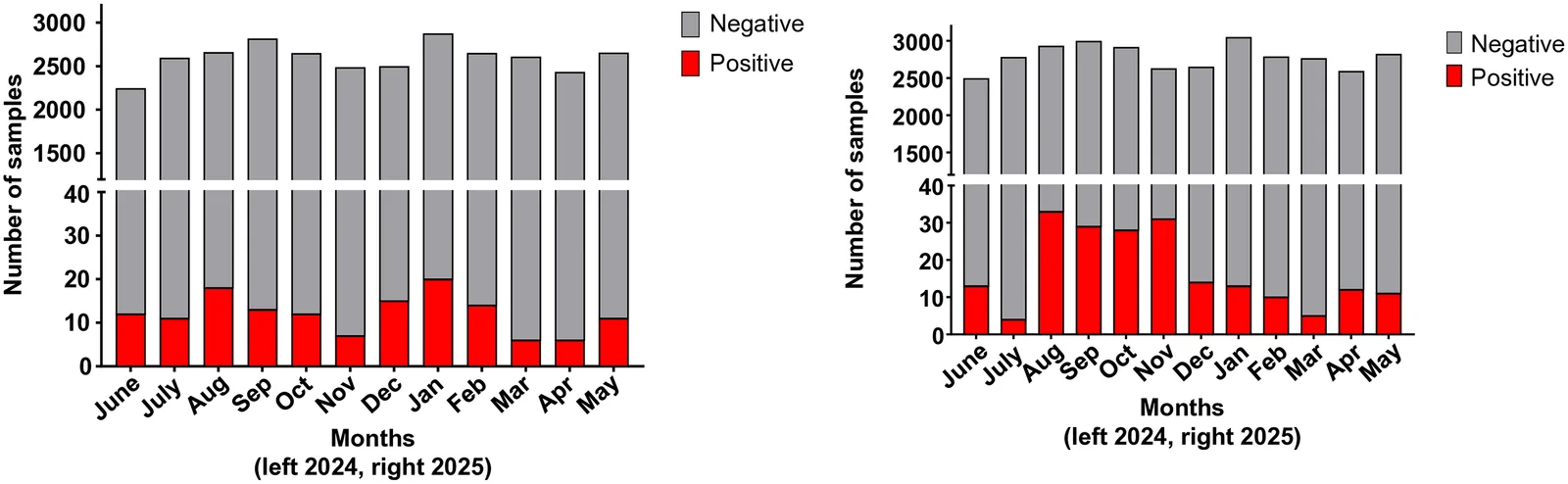
Retrospective laboratory surveillance data on molecular detection of *Cyclospora cayetanensis* (1A) and *Cryptosporidium* spp. (1B). Molecular detection of *Cyclospora cayetanensis* (left) and *Cryptosporidium* spp. (right) in the Netherlands and Curaçao. Positive findings (red) are depicted relative to the total number of fecal samples analysed in the participating laboratories (grey). Results are derived from primary health care (general practitioners) and secondary / tertiary health care (clinical) patients. Data was submitted by five laboratories (*Cyclospora cayetanensis*) and six laboratories (*Cryptosporidium* spp.).

Although exploratory, these findings demonstrate the feasibility of using aggregated routine molecular laboratory data to monitor trends in intestinal protozoal infections across decentralized diagnostic networks.

## Discussion

This nationwide interlaboratory comparison demonstrated good overall interlaboratory agreement for routine molecular detection of *C. cayetanensis* and robust molecular detection of *Cryptosporidium* spp. across decentralized ISO15189-accredited diagnostic laboratories in the Netherlands and Curaçao.

The combination of *Cryptosporidium* spp and *Cyclospora cayetanensis* in one interlaboratory comparison panel provides the possibility to compare the laboratory-specific performances (both parasites) and pathogen-specific assay. Few discordances were seen in the core samples, suggesting robust laboratory processes. Comparison of the workflows that detected Sample 11 with those that did not showed no clear association between successful detection and a specific pre-analytical approach. In particular, mechanical disruption (bead-beating) was not consistently associated with detection of this low-positive sample or with perfect overall performance (S1 Table).

At the pathogen-specific level, the complete qualitative agreement observed for *Cryptosporidium* spp. is reassuring given the substantial methodological heterogeneity between participating laboratories (S1 Table). Laboratories used diverse extraction procedures, amplification platforms, commercial multiplex assays, and laboratory-developed PCRs, yet all workflows correctly identified *Cryptosporidium*-positive and -negative specimens. These findings suggest that contemporary decentralized molecular parasitology diagnostics can achieve robust qualitative performance despite considerable workflow diversity.

In contrast, detection of *C. cayetanensis* showed somewhat more variability, particularly for samples near assay-specific limits of detection. Variability in low-positive *C. cayetanensis* samples likely reflects combined effects of extraction efficiency, sample heterogeneity, stochastic amplification at low target concentrations, assay-specific analytical sensitivity, and differences in Ct interpretation thresholds. Indeed, little variation was seen in laboratories using (almost) same workflows, i.e., workflows number 7–9, showing that standardisation is possible if desired. Most discrepancies occurred in samples with high Ct values, emphasizing the challenges associated with reproducible detection near analytical limits of detection.

These findings are clinically relevant because increasing implementation of multiplex gastrointestinal molecular diagnostics is likely resulting in more frequent detection of low-positive *C. cayetanensis* samples that historically would have remained undetected by microscopy. However, the clinical significance of weak positive *C. cayetanensis* PCR results remains uncertain. High-Ct detections may represent low oocyst density, intermittent shedding, resolving infection, or stochastic amplification near the analytical detection limit [8]. The present study was not designed to determine clinical relevance of such findings, but our results demonstrate that reproducibility decreases for low-positive samples. Weak-positive results should therefore be interpreted using locally validated, assay-specific criteria and in the appropriate clinical context. Given the considerable heterogeneity between workflows, a universal Ct cut-off cannot be recommended.

The use of very broad syndromic gastrointestinal panels has increased the efficiency of diagnostic laboratories and may contribute to detection of more gastrointestinal infections [13], though laboratories should continue critical appraisal of the included pathogens [14,15].

Our findings also underscore the limitations of direct Ct-value comparison between laboratories (S3A-C Tables). Ct values are influenced by multiple workflow-dependent variables, including specimen input, nucleic acid extraction efficiency, amplification chemistry, fluorescence threshold algorithms, and assay design [5–8]. Consequently, Ct values derived from heterogeneous workflows should be interpreted cautiously and should not be considered directly interchangeable between laboratories [7].

An important practical finding was the feasibility of distributing eNAT-preserved stool suspensions under ambient transport conditions. Minimal Ct variation was observed during transport simulations. This supports the use of eNAT-preserved fecal specimens for future decentralized EQA initiatives and multicentre molecular parasitology studies.

The retrospective laboratory data additionally demonstrated the potential utility of routine molecular diagnostic data for decentralized public health surveillance [16]. While *C. cayetanensis* positivity remained low throughout the year, *Cryptosporidium* spp. positivity increased during late summer and autumn, consistent with previously reported seasonal patterns in Europe. Although exploratory, these findings illustrate the potential value of anonymized laboratory network data for monitoring trends in intestinal protozoal infections [17]. However, these aggregated data may be influenced by differences in testing volume, patient population, diagnostic indications, geographic coverage, and laboratory practices, and should therefore not be interpreted as formal prevalence estimates.

This study has several limitations. First, the number of participating laboratories and analysed specimens was relatively limited. Second, participating laboratories used heterogeneous workflows, preventing direct analytical comparison between assays. Third, most specimens lacked detailed clinical metadata and orthogonal confirmatory testing. Fourth, only a limited number of low-positive samples were included, restricting detailed assessment of assay reproducibility near analytical detection limits. Finally, the retrospective surveillance component was exploratory and not designed for formal epidemiological analysis.

In conclusion, routine molecular detection of *C. cayetanensis* demonstrated good overall interlaboratory agreement across decentralized ISO15189-accredited diagnostic laboratories (94.2%; 65/69 core results). However, reproducibility decreased for low-positive samples near assay-specific limits of detection, highlighting the importance of continued assay validation, external quality assessment, and cautious interpretation of weak positive *Cyclospora* PCR results in routine clinical microbiology. These findings support continued development of structured quality assurance initiatives for molecular parasitology diagnostics in decentralized laboratory networks.

## Supporting information

S1 Table Schematic overview of the laboratory-specific workflow applied to the interlaboratory panel. (DOCX)

S2A-C Tables Raw and input-corrected Ct values reported by participants of the interlaboratory comparison. Raw and input-corrected Ct values are depicted for *Cyclospora cayetanensis* (2A,2B) and *Cryptosporidium* spp. (2C).
(DOCX)

S3 Table Primers and probes used by participants of the interlaboratory comparison. (DOCX)

S1A Fig Histogram of historical Ct values observed at the organizing centre. The histogram depicts the distribution of Ct values found for *Cyclospora cayetanensis* by the organizing centre during the period June 2024 – July 2025. Arrows depict the relative position of the selected Ct values in this interlaboratory comparison.
(TIF)

S1B Fig Histogram of historical Ct values observed at the organizing centre. The histogram depicts the distribution of Ct values found for *Cryptosporidium* spp. (S1B) by the organizing centre during the period June 2024 – July 2025. Arrows depict the relative position of the selected Ct values in this interlaboratory comparison.
(TIF)

S2 Fig Comparison of Ct-values at day 0 and day + 8 to +11 for fecal pathogens resuspended in eNAT (Copan). Stability of fecal suspensions in eNAT (Copan) was validated for long term storage at ambient temperature (15 – 25^o^C) using various gastrointestinal parasites: *Cryptosporidium* spp. (N = 3, black), *Blastocystis spp.* (N = 1, green), *Dientamoeba fragilis* (N = 7, orange), *Giardia lamblia* (N = 5, blue). Ct values are based on the Allplex GI-parasite assay (Seegene).
(TIF)

S1 File Questionnaire inclusivity. (DOCX)
