# Supplementary material for: Interlaboratory comparison of molecular detection of Cyclospora cayetanensis in routine diagnostic laboratories
Source: PLoS One. 2026 Oct 28;21(10):e0361578. doi: 10.1371/journal.pone.0361578 (PMC13716187; doi:10.1371/journal.pone.0361578)
Supplement: Supplementary file 1 [file pone.0361578.s001.docx]

#### S1 Table: Schematic overview of the workflows applied to the interlaboratory panel

| Workflow number | Test | Sample input | Suspension volume and pretreatment | Input volume extraction | Output eluate | Input PCR | Score | | Approximate amount of faeces per test and Ct correction factor for faeces (f) or eNAT suspension (e) | |
| --- | --- | --- | --- | --- | --- | --- | --- | --- | --- | --- |
|  |  |  |  |  |  |  | Cyclosp. | Cryptosp. |  |  |
| 1 | BioFire FilmArray gastrointestinal panel (1) | ‘discoloration swab’ | Carry Blair | X | X | X | 1 (FN)  1(FN-ed) | 0 | Unknown | ^A^ |
| 2 | Laboratory-developed PCR (2) | 100 mg | 180 μL ATL + 20 μL proteinase K, o/n 56 ^o^C; QIAmp mini kit | 200 μL | 200 μL | 5 μL | N/A | 0 | 2.5 mg | -2.49 Ct (f)  -1.51 Ct (e) |
| 3 | Laboratory-developed PCR (see supplemental table ) | Pea | 800 μL lysis buffer  o/n freeze/thaw, bead-beating | 200 μL | 50 μL | 5 μL | N/A | 0 | 10 mg | -0.49 Ct (f)  +0.49 Ct (e) |
| 4 | Laboratory-developed PCR (3, 4) | Half pea (10-150 mg), or 200 μL fluid | 1.25 mL STAR – beads, Vortexing | 200 μL | 100 μL | 10 μL | 1 (FP)  1(FN-ed) | 0 | 5 mg | – 1.49 Ct (f)  +0.49 Ct (e) |
| 5 | Laboratory-developed PCR (3, 4) | 200 μL fluid | STAR buffer | 200 μL | 140 μL | 10 μL | 0 ^B^ | 0 | 14 mg | reference |
| 6 | QiaStat GI panel 2 | Half Copan swab or 100 μL fluid | 2 mL Cary-Blair | 200 μL | X | X | 0, did not test ed#11 | 0 | Unknown | ^A^ |
| 7 | STARmag Universal, Allplex GI parasite (Seegene) | Small pea | 2 mL eNAT | 200 μL | 100 μL | 5 μL | 1 (FN)  1(FN-ed) | 0 | 0.5 mg | -5.81 Ct (f)  -0.51 Ct (e) |
| 8 | STARmag Universal, Allplex GI parasite (Seegene) | 2 peas / 4 drops | 2 mL eNAT | 200 μL | 100 μL | 5 μL | 1 (FN)  1(FN-ed) | 0 | 0.5 mg | -4.81 Ct (f)  -0.51 Ct (e) |
| 9 | STARmag Universal, Allplex GI parasite (Seegene) | 2 peas / 4 drops | 2 mL eNAT | 200 μL | 100 μL | 5 μL | 1 (FN)  1(FN-ed) | 0 | 0.5 mg | -4.81 Ct (f)  -0.51 Ct (e) |
| 10 | STARmag Universal, laboratory-developed PCR (3), adapted from (5) | 2 peas / 4 drops | 2 mL eNAT | 200 μL | 100 μL | 10 μL | 0 | 0 | 1 mg | -3.81 Ct (f)  +0.49 Ct (e) |

Weight of pea ~100 mg (personal experience), Corr: Ct correction factor based on input relative to highest input (~14 mg/PCR reaction); calculated as Log(input/reference-input (i.e. 14mg/mL))/Log(2); if the Ct value only depends on the amount of input material per test reaction, the Ct value will be normalized.

Score Cyclosp. (*Cyclospora cayetanensis*) / Score Cryptosp. (*Cryptosporidium spp*) is based on 1 point per false-positive (FP) or false-negative (FN) result. FN-ed denotes a false-negative result on the educational sample (Sample #11).

^A^ Corrections cannot be applied to the BioFire (No known input mass and no Ct value) and the QiaSTAT GI Panel (no known input mass).

^B^ Shown are technical scores based on the PCR, not on reporting. One laboratory (workflow number 5) applied a cut-off of Ct 35.0 for *Cyclospora cayetanensis*, meaning that the educational sample (number 11) would have been reported negative, despite the positive PCR. No other laboratory-developed PCRs applied a cut-off. For commercial platforms without user-accessible Ct values, results were interpreted according to the manufacturer-defined qualitative criteria.

# References

1.	Machiels JD, Cremers AJH, van Bergen-Verkuyten M, Paardekoper-Strijbosch SJM, Frijns KCJ, Wertheim HFL, et al. Impact of the BioFire FilmArray gastrointestinal panel on patient care and infection control. PLoS One. 2020;15(2):e0228596.

2.	Verweij JJ, Blange RA, Templeton K, Schinkel J, Brienen EA, van Rooyen MA, et al. Simultaneous detection of Entamoeba histolytica, Giardia lamblia, and Cryptosporidium parvum in fecal samples by using multiplex real-time PCR. J Clin Microbiol. 2004;42(3):1220-3.

3.	Verweij JJ, Laeijendecker D, Brienen EA, van Lieshout L, Polderman AM. Detection of Cyclospora cayetanensis in travellers returning from the tropics and subtropics using microscopy and real-time PCR. Int J Med Microbiol. 2003;293(2-3):199-202.

4.	Jothikumar N, da Silva AJ, Moura I, Qvarnstrom Y, Hill VR. Detection and differentiation of Cryptosporidium hominis and Cryptosporidium parvum by dual TaqMan assays. J Med Microbiol. 2008;57(Pt 9):1099-105.

5.	Bruijnesteijn van Coppenraet LE, Wallinga JA, Ruijs GJ, Bruins MJ, Verweij JJ. Parasitological diagnosis combining an internally controlled real-time PCR assay for the detection of four protozoa in stool samples with a testing algorithm for microscopy. Clin Microbiol Infect. 2009;15(9):869-74.
