# Supplementary material for: Interlaboratory comparison of molecular detection of Cyclospora cayetanensis in routine diagnostic laboratories
Source: PLoS One. 2026 Oct 28;21(10):e0361578. doi: 10.1371/journal.pone.0361578 (PMC13716187; doi:10.1371/journal.pone.0361578)
Supplement: Supplementary file 2 [file pone.0361578.s002.docx]

**S2A Table: Raw Ct values for *Cyclospora cayetanensis* PCR reported by participants of the interlaboratory comparison.**

| Sample number  Workflow number | 1 | 2 | 3 | 4 | 6 | 7 | 8 |
| --- | --- | --- | --- | --- | --- | --- | --- |
| 4 | 30.68 | 26.82 | 34.80 | 32.16 | 30.71 | 28.02 | 29.03 |
| 5 * | 32.9 | 32.3 | 32.9 | 32.7 | 31.5 | 32.5 | 31.6 |
| 6 | 28.32 | 30.68 | 30.77 | 27.79 | 27.59 | 25.11 | 28.55 |
| 7 | 38.49 | 38.94 | 38.29 | X | 37.79 | 39.52 | 39.85 |
| 8 | 38.44 | 39.51 | 39.80 | 40.51 | 40.78 | X | 38.60 |
| 9 | 37.86 | 38.36 | 39.49 | 42.65 | 36.67 | X | 38.65 |
| 10 | 32.16 | 32.65 | 34.07 | 35.27 | 32.34 | 33.70 | 33.15 |

x = negative. Sample 1,2,8: unpreserved stool sample. Sample 3,4,6,7: stool suspension in eNAT at approximately 100mg/mL. Details of the workflows can be found in S1 Table. * reference laboratory for Ct value correction.

**S2B Table: Corrected Ct values for *Cyclospora cayetanensis* PCR reported by participants of the interlaboratory comparison.**

| Sample number  Workflow number | 1 | 2 | 3 | 4 | 6 | 7 | 8 |
| --- | --- | --- | --- | --- | --- | --- | --- |
| 4 | 29.19 | 25.33 | 35.29 | 32.65 | 31.2 | 28.51 | 27.54 |
| 5 * | 32.9 | 32.3 | 32.9 | 32.7 | 31.5 | 32.5 | 31.6 |
| 6 | ǂ | ǂ | ǂ | ǂ | ǂ | ǂ | ǂ |
| 7 | 32.68 | 33.13 | 37.78 | X | 37.28 | 39.01 | 34.04 |
| 8 | 33.63 | 34.7 | 39.29 | 40 | 40.27 | X | 33.79 |
| 9 | 34.05 | 34.55 | 39.98 | 43.14 | 37.16 | X | 34.84 |
| 10 | 27.35 | 27.84 | 33.56 | 34.76 | 31.83 | 33.19 | 28.34 |

N/A = not conducted, x = negative. Sample 1,2,8: unpreserved stool sample. Sample 3,4,6,7: stool suspension in eNAT at approximately 100mg/mL. Ct correction was based on the amount of the unpreserved stool or eNAT suspension per PCR test; dilution: amount in test / amount in reference test. Correction; Raw Ct value - Log(dilution)/Log(2). * reference laboratory for Ct value correction. ǂ : no input available, therefore no correction can be applied. Details of the workflows can be found in S1 Table.

**S2C Table: Raw and Corrected Ct values for *Cryptosporidium spp* PCR reported by participants of the interlaboratory comparison.**

|  | raw Ct value | | |  | Input corrected Ct-value | | | |
| --- | --- | --- | --- | --- | --- | --- | --- | --- |
| Sample number  Workflow number | 5 | 9 | 10 |  | 5 | 9 | 10 |  |
| 2 | 30 | 35 | 28 |  | 28.49 | 33.49 | 26.49 |  |
| 3 | 27.9 | 34.4 | 26.7 |  | 28.39 | 34.89 | 27.19 |  |
| 4 | 28.27 | 33.80 | 31.30 |  | 28.76 | 34.29 | 31.79 |  |
| 5 * | 27.5 | 30.4 | 28.8 |  | 27.5 | 30.4 | 28.8 |  |
| 6 | 20.86 | N/A | 20.87 |  | ǂ | ǂ | ǂ |  |
| 7 | 32.19 | 38.12 | 33.79 |  | 31.68 | 37.61 | 33.28 |  |
| 8 | 32.32 | 36.63 | 34.11 |  | 31.81 | 36.12 | 33.6 |  |
| 9 | 33.30 | 37.38 | 33.79 |  | 33.79 | 37.87 | 34.28 |  |
| 10 | 32.09 | 36.30 | 32.44 |  | 31.58 | 35.79 | 31.93 |  |

N/A = not conducted. Sample 5,9,10: stool suspension in eNAT at approximately 100mg/mL. Ct correction was based on the amount of eNAT suspension per PCR test; dilution: amount in test / amount in reference test. Correction; Raw Ct value - Log(dilution)/Log(2). * reference laboratory for Ct value correction. ǂ : no input available, therefore no correction can be applied. Details of the workflows can be found in S1 Table.
