# Supplementary material for: Interlaboratory comparison of molecular detection of Cyclospora cayetanensis in routine diagnostic laboratories
Source: PLoS One. 2026 Oct 28;21(10):e0361578. doi: 10.1371/journal.pone.0361578 (PMC13716187; doi:10.1371/journal.pone.0361578)
Supplement: Supplementary file 3 [file pone.0361578.s003.docx]

#### S3 Table: Primers and probes used by participants of the interlaboratory comparison

| *Cyclospora cayetanensis* | |  |  |
| --- | --- | --- | --- |
| Forward | Reverse | Probe | Reference |
| TAGTTAACCGAACGGATCGCATT | AATGCCACGTAGGCCAATA | Q705 CGGCGATAGATCATTCAAGTTTCTGACC BHQ1 | (1) |
| *Cryptosporidium parvum/hominis* | |  |  |
| Forward | Reverse | Probe | Reference |
| CTTTTTACCAATCACAGARTCATCAGA | YGTGTTTGCCAATGCATATGAA | NED MGB TCGACTGGTATCCCTATAA | Adapted from (2) |
| CGCTTCTCTAGCCTTTCATGA | CTTCACGTGTGTTTGCCAAT | Texas Red CCAATCACAGAATCATCAGAATCGACTGGTATC BHQ2 | (3), based on (4) |
| CATTCAAGTTTCTGACCTATCAGC | TCCCCGTTACCCGTCATWG | Texas Red TTAGACGGTAGGGTATTGGC MGB-EDQ | LDT |
| ATGACGGGTAACGGGGAAT | CCAATTACAAAACCAAAAAGTCC | Cy5 CGCGCCTGCTGCCTTCCTTAGATG BHQ (*Cryptosporidium* spp.) | (5) |
| ACTTTTTGTTTGTTTTACG CCG | AATGTGGTAGTTGCGGTTGAA | FAM ATTTATTAATTTATCTCTTACTTCGT BHQ (*C. hominis*) |  |
| ACTTTTTGTTTGTTTTACGCCG | AATGTGGTAGTTGCGGTTGAA | FAM ATTTATCTCTTCGTAGCGGCG BHQ (*C. parvum*) |  |

LDT: laboratory-developed assay.

# **References**

1.	Verweij JJ, Laeijendecker D, Brienen EA, van Lieshout L, Polderman AM. Detection of Cyclospora cayetanensis in travellers returning from the tropics and subtropics using microscopy and real-time PCR. Int J Med Microbiol. 2003;293(2-3):199-202.2.	Bruijnesteijn van Coppenraet LE, Wallinga JA, Ruijs GJ, Bruins MJ, Verweij JJ. Parasitological diagnosis combining an internally controlled real-time PCR assay for the detection of four protozoa in stool samples with a testing algorithm for microscopy. Clin Microbiol Infect. 2009;15(9):869-74.

3.	Verweij JJ, Blange RA, Templeton K, Schinkel J, Brienen EA, van Rooyen MA, et al. Simultaneous detection of Entamoeba histolytica, Giardia lamblia, and Cryptosporidium parvum in fecal samples by using multiplex real-time PCR. J Clin Microbiol. 2004;42(3):1220-3.

4.	Fontaine M, Guillot E. Development of a TaqMan quantitative PCR assay specific for Cryptosporidium parvum. FEMS Microbiol Lett. 2002;214(1):13-7.

5.	Jothikumar N, da Silva AJ, Moura I, Qvarnstrom Y, Hill VR. Detection and differentiation of Cryptosporidium hominis and Cryptosporidium parvum by dual TaqMan assays. J Med Microbiol. 2008;57(Pt 9):1099-105.
